# Galactose protects against cell damage in mouse models of acute pancreatitis

**DOI:** 10.1172/JCI94714

**Published:** 2018-07-30

**Authors:** Shuang Peng, Julia V. Gerasimenko, Tetyana M. Tsugorka, Oleksiy Gryshchenko, Sujith Samarasinghe, Ole H. Petersen, Oleg V. Gerasimenko

**Affiliations:** 1 Cardiff School of Biosciences, Cardiff University, Cardiff, United Kingdom.; 2 Department of Physiology, School of Medicine, Jinan University, Guangzhou, China.; 3 Bogomoletz Institute of Physiology, Kiev, Ukraine.; 4 Great Ormond Street Hospital for Children, NHS Foundation Trust, London, United Kingdom.

**Keywords:** Gastroenterology, Oncology, Calcium signaling, Leukemias

## Abstract

Acute pancreatitis (AP), a human disease in which the pancreas digests itself, has substantial mortality with no specific therapy. The major causes of AP are alcohol abuse and gallstone complications, but it also occurs as an important side effect of the standard asparaginase-based therapy for childhood acute lymphoblastic leukemia. Previous investigations into the mechanisms underlying pancreatic acinar cell death induced by alcohol metabolites, bile acids, or asparaginase indicated that loss of intracellular ATP generation is an important factor. We now report that, in isolated mouse pancreatic acinar cells or cell clusters, removal of extracellular glucose had little effect on this ATP loss, suggesting that glucose metabolism was severely inhibited under these conditions. Surprisingly, we show that replacing glucose with galactose prevented or markedly reduced the loss of ATP and any subsequent necrosis. Addition of pyruvate had a similar protective effect. We also studied the effect of galactose in vivo in mouse models of AP induced either by a combination of fatty acids and ethanol or asparaginase. In both cases, galactose markedly reduced acinar necrosis and inflammation. Based on these data, we suggest that galactose feeding may be used to protect against AP.

## Graphical abstract

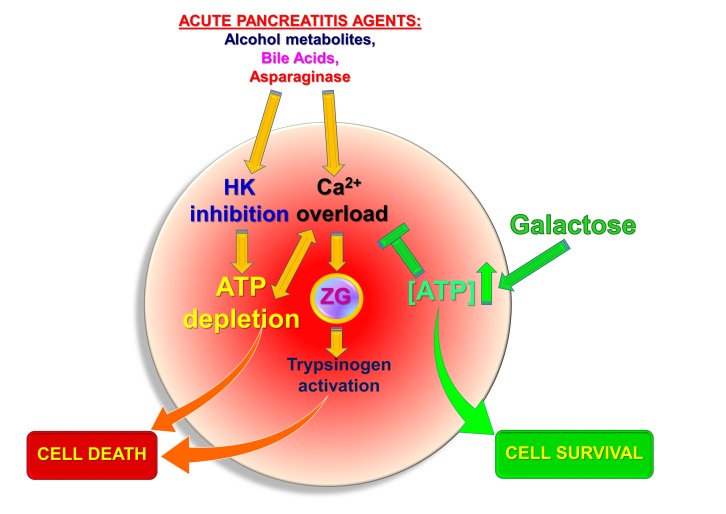

## Introduction

Acute pancreatitis (AP) is an inflammatory disease that originates in the exocrine pancreas, where inactive pancreatic proenzymes become prematurely activated inside the pancreatic acinar cells (PACs), digesting the pancreas and its surroundings (1, 2). The main causes of AP are excessive alcohol and fatty food intake and gallstone disease, accounting for about 80% of all cases (3). Stimulation of PACs with alcohol metabolites or bile acids (BAs) leads to aberrant calcium signaling due to excessive release from intracellular stores, followed by activation of massive Ca^2+^ entry through store-operated Ca^2+^ release–activated Ca^2+^ (CRAC) channels, causing intracellular Ca^2+^ overload (2, 4, 5).

Another cause of AP is the l-asparaginase treatment of acute lymphoblastic leukemia (ALL) (6, 7). According to Cancer Research UK, there were 832 new cases of ALL diagnosed in the United Kingdom in 2015. The incidence rates for ALL are highest in children aged 0 to 4 (2012–2014). Antileukemic drugs based on l-asparaginase are currently used in the clinic as an effective treatment for childhood ALL (8–12). However, in up to 10% of cases, the asparaginase treatment has to be truncated due to development of AP, a serious and incurable illness (6, 7, 13–17). Although asparaginase-based drugs have been used in the clinic for many years (8), the mechanism of this side effect has not been well explored and understood.

We have recently made progress in understanding the mechanism of asparaginase-induced AP (AAP) (18). Our key findings include the activation of protease-activated receptor 2 (PAR2) as well as calcium overload and loss of ATP in PACs. We believe these findings provide the first mechanistic insight into the process by which asparaginase treatment of ALL may cause AAP. The asparaginase effect on cancer cells relies on the depletion of asparagine, which the malignant cells cannot produce by themselves, as opposed to normal cells (19, 20). However, the AP-inducing side effects of asparaginase do not depend on the presence or absence of asparagine (18). In contrast, the AP-inducing side effect of asparaginase is caused by the activation of a signal transduction mechanism involving PAR2 that, via a number of steps, causes cytosolic Ca^2+^ overloading and reduction in intracellular ATP levels. The reduction of energy supply inhibits both the plasma membrane Ca^2+^ ATPase (PMCA) and the sarco/endoplasmic reticulum Ca^2+^ ATPase (SERCA) (21–23). We have recently shown that restoration of energy supply, by the addition of pyruvate, provides an astonishingly high degree of protection against pancreatic necrosis (18).

We have now analyzed the role of glycolysis in AP in more detail, in vivo and in vitro, and specifically compared the effects of pyruvate, galactose (24), and glucose on the functional and morphological features of AP and AAP. Based on these data, we propose a simple and promising way to rescue intracellular ATP levels in AP and AAP patients.

## Results

### ATP loss is the common hallmark of AP.

**Figure 1:**
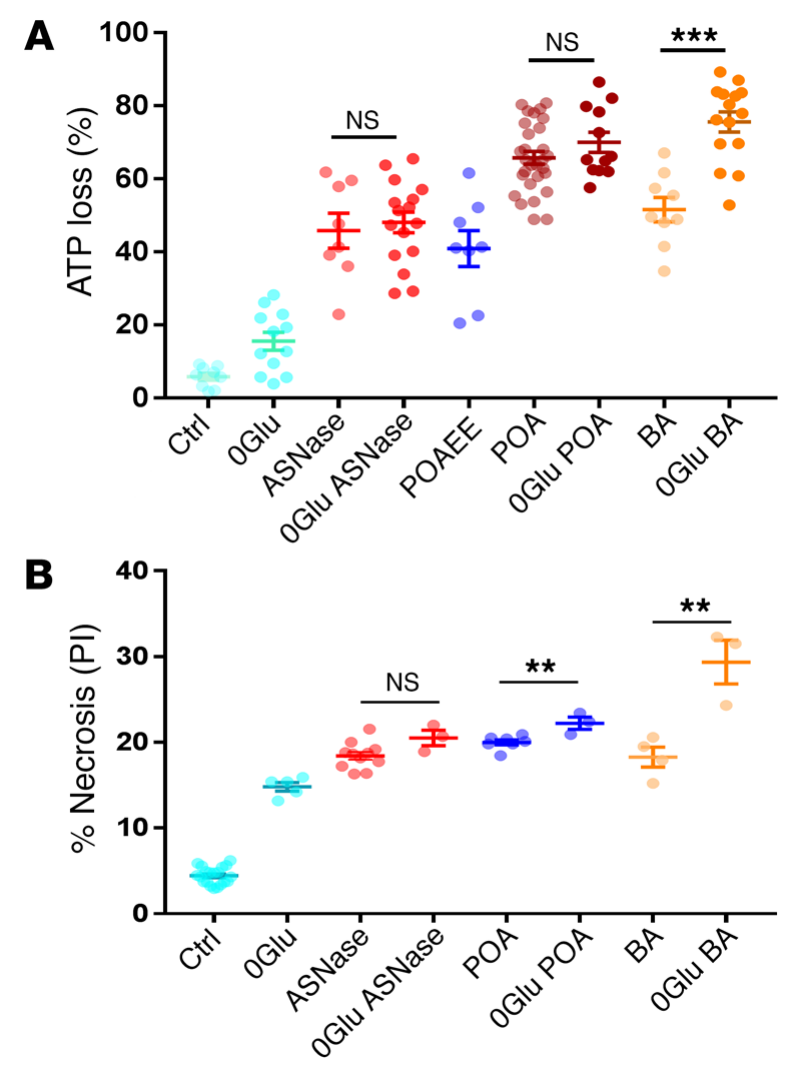
Asparaginase, POAEE with POA, and BAs all induce substantial ATP loss and cell necrosis. (**A**) Comparison of cellular ATP depletion in PACs after treatment of cells for 30 minutes with or without glucose (0Glu), or application of asparaginase (ASNase), POAEE, POA, or BA. Level of ATP loss (measured by MgGreen) is shown as a percentage of full depletion by a mixture of CCCP, oligomycin, and iodoacetate. Dots represent ATP loss (%) in each cell. Data are shown as mean ± SEM. Ctrl, control. (**B**) Summary of cell necrosis measurements in PACs treated with asparaginase, POA, or BA for 2 hours in the presence or absence of 10 mM glucose as compared with control. Removal of glucose had little effect on asparaginase and POA, but increased BA-induced necrosis. Cells were stained with PI. Dots represent series of experiments with *n* > 100 cells in each sample. Data are shown as mean ± SEM. **P < 0.01; ***P < 0.001, 1-way ANOVA.

It has been established previously that ATP loss in AP is a critical part of the pathological mechanism in PACs, irrespective of whether it has been initiated by alcohol metabolites or BAs (1, 22, 25). As previously described (18), we have assessed intracellular changes in ATP concentration by using Magnesium Green (MgGreen) fluorescence measurements. As most of the intracellular ATP will be in the form of Mg-ATP, a reduction of the ATP concentration will increase the fluorescence intensity of MgGreen due to the increase in free Mg^2+^ concentration. We have studied the effect of asparaginase in PACs and found that 30 minutes of exposure to this agent caused a 45.8% ± 4.8% loss of ATP (Figure 1A).

The ATP reduction induced by asparaginase was very similar to that elicited by exposure to the nonoxidative alcohol metabolite palmitoleic acid ethyl ester (POAEE) (40.9% ± 4.9%) and palmitoleic acid (POA) (66.9% ± 4.9%) (26) or a BA mixture (51.6% ± 3.3%) (Figure 1A), while removal of glucose for 30 minutes led to a substantially smaller reduction (15.5% ± 0.95%). Interestingly, removal of glucose did not significantly increase ATP depletion induced by POA or ASNase, but partially increased ATP depletion induced by BA (Figure 1A).

Since the majority of cellular ATP is produced by glucose metabolism, we compared the effect of a glucose-free medium on necrosis to that induced by asparaginase, POAEE, POA, or BA (Figure 1B). In these experiments, lasting 2 hours, we found that removal of glucose produced a level of necrosis comparable to that of all other pathological agents (14.8% ± 0.5%, *P* < 0.0001), but did not significantly exacerbate the effects of asparaginase (*P* > 0.059). It only marginally increased POA-elicited necrosis (from 20.0% ± 0.3% to 22.2% ± 0.7%, *P* < 0.01) (Figure 1B) and somewhat increased BA-induced necrosis (from 18.3% ± 1.1% to 29.4 ± 2.5%, *P* < 0.008). The fact that removal of glucose did not further increase the extent of necrosis induced by asparaginase or POA may suggest that glucose metabolism is already so strongly inhibited by these 2 agents that removal of external glucose has practically no additional effect.

### Pyruvate and galactose alleviate bile- and alcohol metabolite–induced pathology.

**Figure 2:**
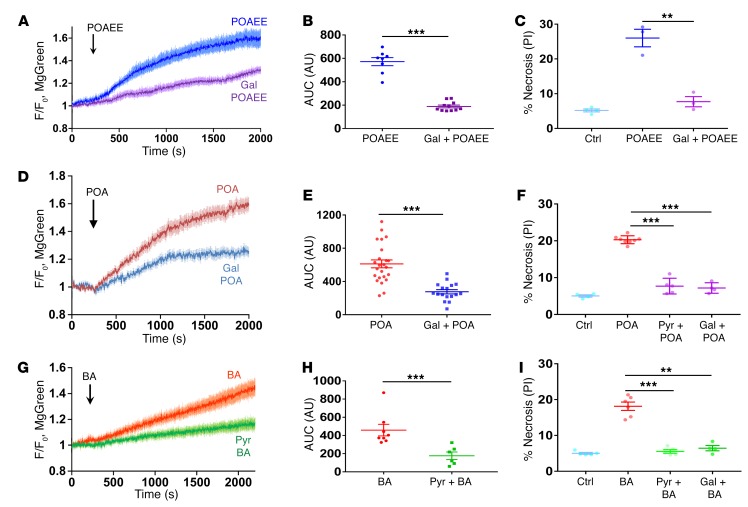
Pyruvate and galactose provide substantial protection against alcohol- and bile-induced ATP loss and necrosis in PACs. (**A**) POAEE-induced (500 μM) ATP depletion is markedly reduced by adding 1 mM galactose (Gal). Averaged normalized (F/F_0_) traces with error bars (POAEE, *n* = 8; galactose + POAEE, *n* = 11). (**B**) AUC comparison of traces shown in **A**. Galactose-reduced POAEE-induced ATP depletion (*P* < 0.0001, 2-tailed Student’s *t* test). (**C**) POAEE-induced necrosis was significantly reduced by adding 1 mM galactose (*P* < 0.003, 1-way ANOVA; 3 series of experiments with more than 100 cells in each sample). (**D**) POA-induced (50 μM) ATP depletion is reduced by replacing glucose with 10 mM galactose. Averaged traces with error bars (POA, *n* = 24; galactose + POA, *n* = 17). (**E**) AUC comparison of traces shown in **D**. Galactose significantly reduced POA-induced ATP depletion (*P* < 0.0001, 2-tailed Student’s *t* test). (**F**) POA-induced necrosis was reduced significantly by replacing glucose with either 10 mM pyruvate (Pyr) or 10 mM galactose (*P* < 0.0001 in both series, 1-way ANOVA; dots represents a series of experiments with more than 100 cells in each sample). (**G**) BA mixture–induced ATP depletion is reduced by adding 1 mM pyruvate. Averaged traces with error bars (BA, *n* = 8; BA + pyruvate, *n* = 6). (**H**) AUC comparison of traces shown in **G**. Pyruvate significantly reduces BA-induced ATP depletion (*P* < 0.002, 2-tailed Student’s *t* test). (**I**) BA-induced necrosis is reduced to nearly control level by replacing glucose with 10 mM pyruvate (*P* < 0.00015, 1-way ANOVA; 5 series of experiments with more than 100 cells in each sample) or 10 mM galactose (*P* < 0.008, 1-way ANOVA; 4 series of experiments with more than 100 cells in each sample). **P < 0.01; ***P < 0.001, 2-tailed Student’s *t* test (**B**, **E**, **H**); 1-way ANOVA (**C**, **F**, **I**).

In our previous study into the mechanism by which asparaginase evokes pathological changes in isolated PACs (18), we showed that inclusion of pyruvate in the bathing solution provided remarkable protection against necrosis. We further demonstrated that the reduction in the intracellular ATP level caused by asparaginase was significantly diminished when pyruvate was present (18). In addition to pyruvate, we decided to test galactose for its effectiveness in protection against alcohol- and bile-induced pancreatic pathologies. Galactose very significantly reduced the ATP loss caused by the alcohol metabolite POAEE (Figure 2, A and B) and POA (Figure 2, D and E) and also essentially prevented the necrosis induced by these agents (Figure 2, C and F). Pyruvate had a very similar effect (Figure 2F). A comparable protective effect of pyruvate was also found in the case of bile-related pathology. Pyruvate substantially reduced the ATP loss elicited by BA (Figure 2, G and H), and both pyruvate and galactose almost entirely eliminated BA-induced necrosis (Figure 2I).

### Pyruvate and galactose protect against asparaginase-induced necrosis.

**Figure 3:**
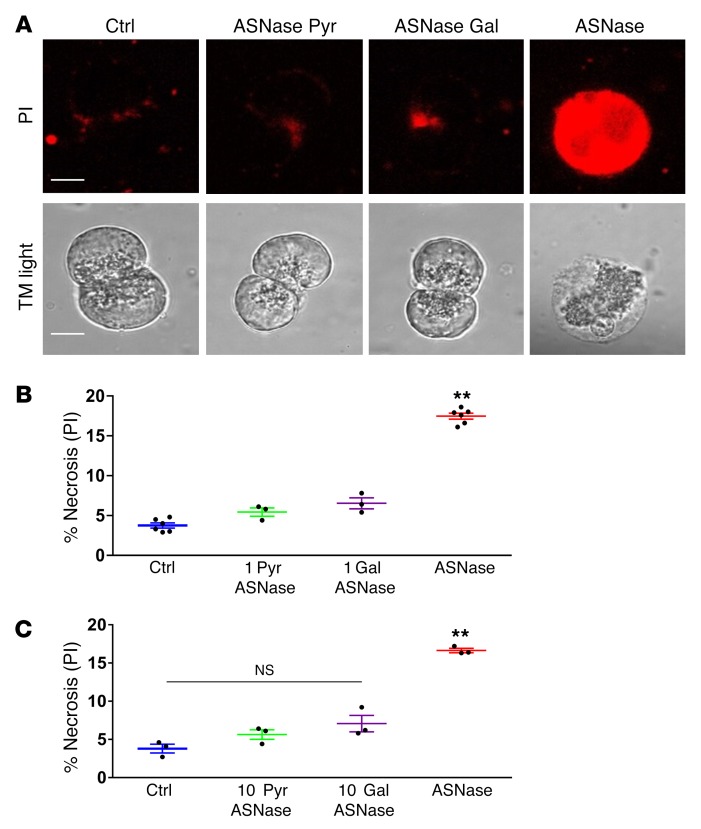
Pyruvate and galactose significantly reduce the level of asparaginase-induced necrosis. (**A**) Representative images of cells from experiments shown in **B** and **C**. Scale bars: 10 μm. (**B**) In PACs, 1 mM pyruvate (5.4% ± 0.5%, 3 series with *n* > 300, *P* < 0.0002) or 1 mM galactose (6.5% ± 0.7%, 3 series with *n* > 300, *P* < 0.0004) reduce the asparaginase-induced necrosis level as compared with asparaginase alone (17.5% ± 0.4%, 6 series with *n* > 300). (**C**) Complete replacement of extracellular glucose (10 mM) with pyruvate (10 mM) (5.6% ± 0.6%, 3 series with *n* > 300, *P* < 0.0001) or 10 mM galactose (7.1% ± 1.1%, 3 series with *n* > 300, *P* < 0.001) significantly reduces the asparaginase-induced necrosis level as compared with the control level (3.8% ± 0.6%, 3 series with *n* > 300). **P < 0.01, 1-way ANOVA.

The ability of galactose to protect against necrosis induced by POAEE, POA, or BA (Figure 2, C, F, and I) has prompted us to also test the effect of galactose on asparaginase-induced pathology (18). Both, pyruvate and galactose, at either 1 mM (Figure 3, A and B) or 10 mM (Figure 3C), had similar protective effects against asparaginase-induced necrosis in PACs. Interestingly, the presence or absence of glucose made no difference in the extent of the necrosis (Figure 1B). These data suggest that glucose metabolism is severely affected by asparaginase, but that energy supply can be replenished by galactose or pyruvate joining the glycolysis cycle.

### Galactose and pyruvate, but not glucose, alleviate asparaginase-induced pathology.

**Figure 4:**
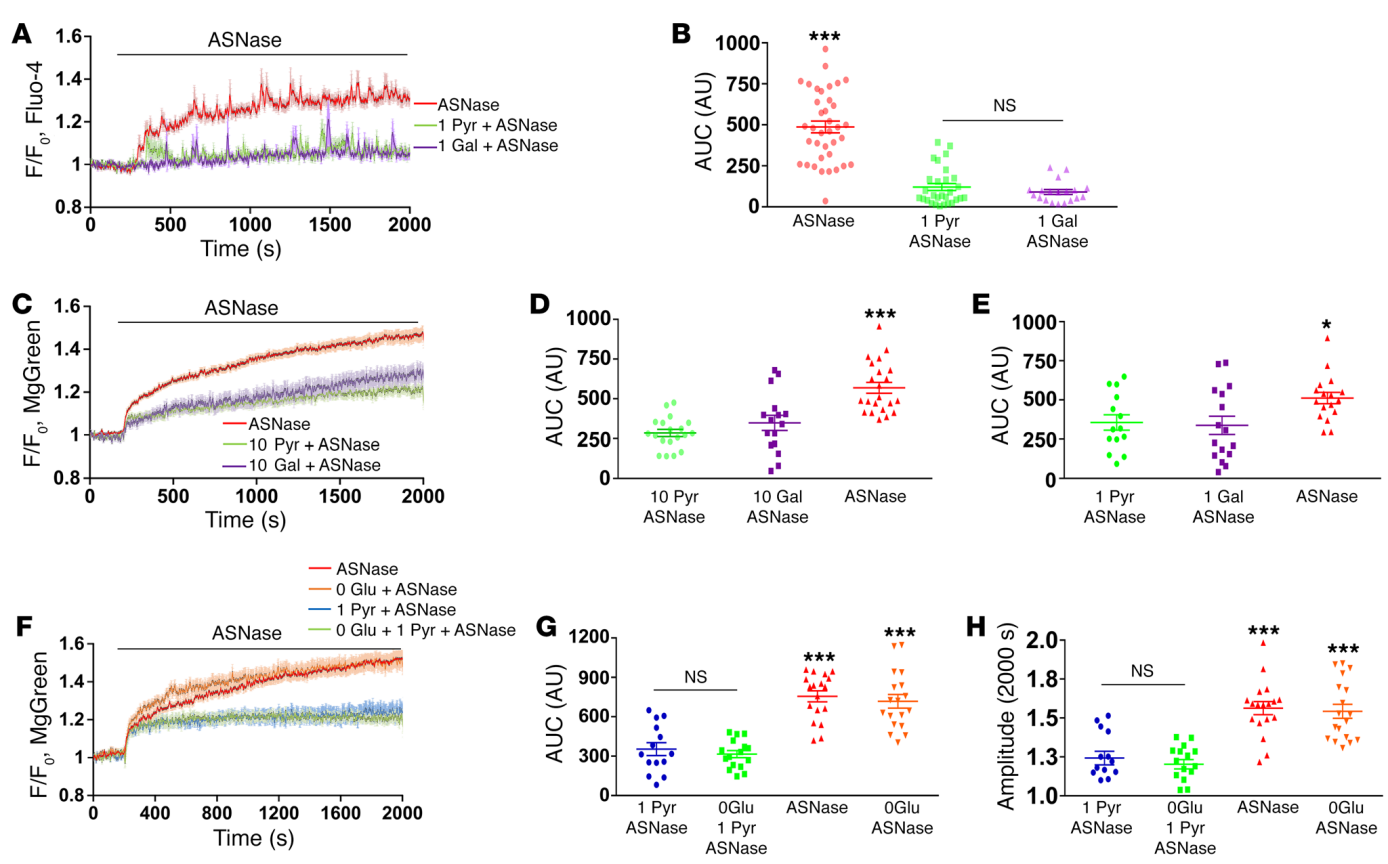
Asparaginase-induced Ca^2+^ overload and ATP loss were substantially reduced by galactose or pyruvate, but not glucose removal. (**A**) Asparaginase elicits elevated [Ca^2+^]_i_ plateau. Averaged traces with error bars shown (red, *n* = 35). Green trace shows reduced response in the presence of 1 mM pyruvate (after 5 minutes of preincubation, *n* = 33). Purple trace shows reduced response in the presence of 1 mM galactose (15 minutes of preincubation, *n* = 17). (**B**) Comparison of AUC shown in **A** during first 30 minutes of [Ca^2+^]_i_ change in the presence of pyruvate (green) and galactose (purple) or asparaginase alone (red) (*P* < 0.0001). (**C**) ATP loss was evaluated using MgGreen. Replacement of extracellular glucose (10 mM) with pyruvate (10 mM) (green, *n* = 19) or galactose (10 mM) (purple, *n* = 16) for 30 minutes markedly reduces [Mg^2+^]_i_ change induced by asparaginase (red, *n* = 38). (**D**) Quantitative analysis of experiments of type shown in **C** (AUC during 30 minutes; *P* < 0.0005). (**E**) Quantitative analysis of experiments as in **C**, but with 1 mM of either pyruvate (green, *n* = 14) or galactose (purple, *n* = 16). Bars show AUC recorded during 30 minutes of asparaginase application (*P* < 0.015). (**F**) Asparaginase induces ATP loss irrespective of glucose presence (red, *n* = 21) or absence (orange, *n* = 17). Pyruvate (1 mM) decreased ATP depletion irrespective of glucose absence (green trace, *n* = 16) or presence (10 mM) (blue trace, *n* = 14). (**G**) Quantitative analysis of experiments shown in **F** by AUC during 30 minutes of asparaginase application. Pyruvate (blue and green) was highly protected against ATP depletion (*P* < 0.0001) regardless of glucose (*P* > 0.05). (**H**) Amplitudes at 2,000 seconds shown in **F**. Pyruvate (blue and green) is protected against ATP depletion; *P* < 0.0001 regardless of presence (red) or absence of glucose (orange). *P < 0.05; ***P < 0.001, 1-way ANOVA.

With regard to the primary action of asparaginase on PACs, we have previously shown that this agent evokes a sustained elevation of cytosolic Ca^2+^ concentration ([Ca^2+^]_i_) due to interaction with PAR2 (18). Figure 4, A and B, shows that both pyruvate and galactose very markedly reduced the increase in asparaginase-elicited [Ca^2+^]_i_. In control experiments, pyruvate and galactose did not change the frequency of Ca^2+^ oscillations induced by either cholecystokinin (CCK) (*P* > 0.3, *n* = 11 and *P* > 0.9, *n* = 11 respectively) or asparaginase (*P* > 0.1, *n* = 33 and *P* > 0.7, *n* = 17, respectively). There was also no significant difference with regard to the amplitude of spikes induced by CCK (*P* > 0.8, *n* = 157 and *P* > 0.8, *n* = 46, respectively). The amplitude of asparaginase-induced oscillations was reduced by 20% (*P* < 0.0001, *n* = 39) in the presence of pyruvate and by 15% (*P* < 0.02, *n* = 26) in the presence of galactose. These relatively minor effects are probably due to the increase in the cytoplasmic ATP level and, therefore, Ca^2+^ uptake after release. Previously, we have shown that asparaginase inhibits Ca^2+^ extrusion from PACs, most likely due to the reduced availability of ATP (18).

Although the asparaginase-elicited sustained elevation of [Ca^2+^]_i_ depends on increased Ca^2+^ entry (18), this could be compensated for by an increase in the rate of active Ca^2+^ extrusion if an adequate supply of ATP were available. It would seem possible that ATP supply is enhanced in the presence of pyruvate or galactose and that this could be the mechanism by which toxic [Ca^2+^]_i_ increase is inhibited. We therefore tested this hypothesis by assessing changes in intracellular ATP concentration (Figure 4, C and D) as well as changes in NADH and flavin adenine dinucleotide (FAD) (Supplemental Figure 1, A and B; supplemental material available online with this article; https://doi.org/10.1172/JCI94714DS1).

Asparaginase induced a substantial intracellular ATP loss (Figure 4, C–E), in line with reduction of NADH (Supplemental Figure 1A). Replacement of glucose with pyruvate or galactose (both 10 mM, Figure 4, C and D) or adding 1 mM pyruvate or galactose (Figure 4E) markedly reduced asparaginase-induced ATP loss. Replacing glucose with pyruvate or galactose was very effective in protecting against ATP loss, and we therefore compared our results for the presence and absence of glucose and pyruvate (Figure 4, F–H). The ATP loss was substantially higher in the absence of pyruvate (red and orange traces) regardless of the presence or absence of glucose. Comparison of the AUC shows that 1 mM pyruvate (blue and green traces) significantly reduced ATP loss (Figure 4G), whereas the presence of 10 mM glucose did not (*P* > 0.05). Comparison of the amplitudes (Figure 4H) showed very similar results, namely that the glucose-independent ATP loss was markedly reduced by 1 mM pyruvate. Asparaginase also affected the mitochondrial potential (Supplemental Figure 2, A–F) and the mitochondrial Ca^2+^ levels (Supplemental Figure 3, A–F), but pyruvate and galactose restored these parameters to near control levels.

### Pyruvate and galactose increase intracellular ATP levels.

**Figure 5:**
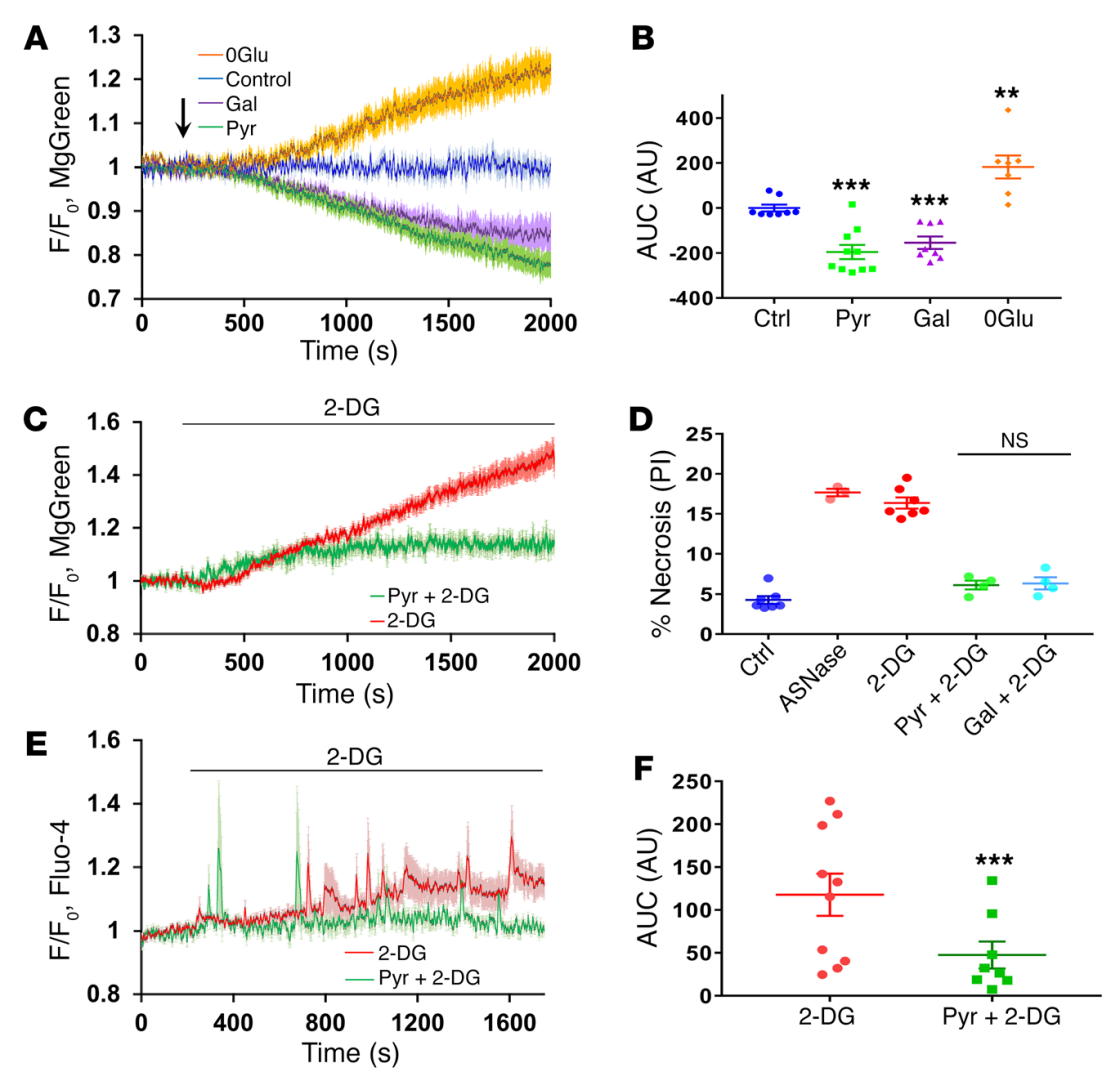
Pyruvate and galactose increase intracellular ATP levels and protect cells against ATP depletion induced by 2-DG. (**A**) Glucose removal induces substantial ATP depletion, whereas pyruvate or galactose boosts ATP production. Average traces show normalized changes of MgGreen fluorescence in PACs in the presence (blue trace, *n* = 8) or absence (orange trace, *n* = 7) of 10 mM glucose or in the presence of pyruvate (1 mM; green trace, *n* = 10) or galactose (1 mM; purple trace, *n* = 8). (**B**) Comparison of AUC for experiments shown in **A**. **P < 0.01; ***P < 0.001, 1-way ANOVA. (**C**) Pyruvate markedly reduces ATP depletion induced by 10 mM 2-DG. Averaged trace (shown with error bars) represents the result of application of 2-DG in the absence (red trace, *n* = 9) or presence of 1 mM pyruvate (after 5 minutes of preincubation, green trace, *n* = 7). (**D**) Comparison of necrotic cell death levels induced by 2 hours of incubation of PACs with 10 mM 2-DG or asparaginase with control (nontreated cells) (PI-stained cells, *P* = 0.36, 1-way ANOVA, 3 series of experiments with *n* > 100 cells in each sample). (**E**) Average traces show normalized changes of Fluo-4 fluorescence in PACs induced by 10 mM 2-DG alone (red trace, *n* = 10) or after 5 minutes preincubation of cells and continuous presence of 1 mM pyruvate (green trace, *n* = 8) for 25 minutes. (**F**) Quantitative analysis of experiments of the type shown in **E** by comparing AUC for 25 minutes of the recording after application of 10 mM 2-DG. ***P < 0.001, 2-tailed Student’s *t* test.

All the 3 AP-inducing factors that we tested substantially inhibited ATP production, and both asparaginase and POA severely inhibited glucose metabolism. Galactose can enter the glycolysis cycle, skipping its first step, and does not depend on hexokinase (HK) activity. Our data may therefore indicate that glucokinase/HK activity is inhibited during the induction of AP. Both galactose and pyruvate provide an additional source of ATP and increase intracellular ATP levels (Figure 5, A and B).

The glucose analogue 2-deoxy-d-glucose (2-DG) (27), which inhibits glycolysis via its indirect actions on HK, induced a substantial ATP loss (Figure 5C), necrosis (Figure 5D), and [Ca^2+^]_i_ elevation (Figure 5, E and F); these effects are very similar to those induced by asparaginase (Figure 4, A–D; Figure 3B; and Figure 5D). Pyruvate significantly reduced the 2-DG–induced sustained [Ca^2+^]_i_ elevation (Figure 5, E and F). The protective effects of galactose were completely blocked by the glucose transport inhibitor phloretin (Supplemental Figure 1C), suggesting that only HK inhibition could explain the ATP depletion observed in AP.

### HK activity is inhibited in vitro by POA and BA.

**Figure 6:**
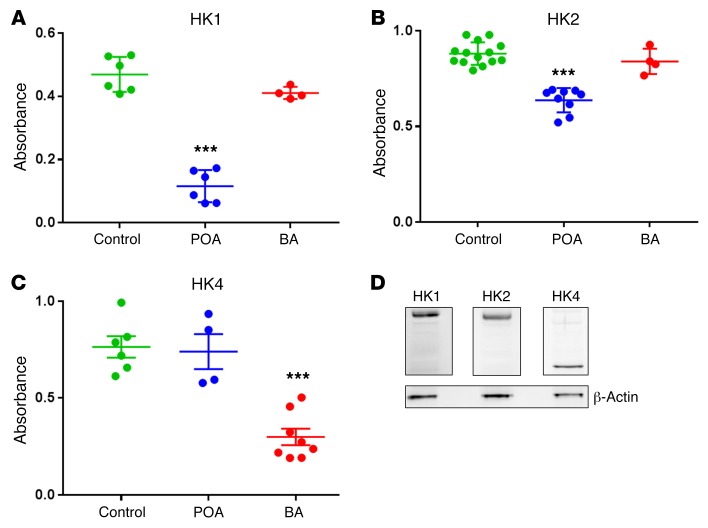
HK activity is significantly inhibited in vitro by POA and BA. (**A**) HK1 activity is reduced significantly by 0.1 mM POA (*n* = 6, *P* < 0.0001), but not changed significantly by 0.05% BA (*n* = 4, *P* > 0.13) as compared with control (*n* = 6). (**B**) HK2 activity is reduced significantly by 0.1 mM POA (*n* = 9, *P* < 0.0001), but not affected by 0.05% BA (*n* = 4, *P* > 0.3) as compared with control (*n* = 13). (**C**) HK4 activity is reduced significantly by 0.05% BA (*n* = 8, *P* < 0.0001), but not affected by 0.1 mM POA (*n* = 4, *P* > 0.8) as compared with control (*n* = 6). (**D**) Western blot analysis of the expression levels of HK1, HK2, and HK4 in PACs (representative case, repeated 3 times with similar results). ***P < 0.001, 1-way ANOVA.

To test our hypothesis that AP-inducing agents cause intracellular ATP loss by reducing HK activity, we measured the activities of the 3 major human HKs present in the pancreas in vitro (28, 29). We found that POA markedly reduced the activity of HK1 and partially reduced HK2 activity (Figure 6, A and B). Whereas POA had no effect on glucokinase (HK4), BA markedly reduced HK4 activity (Figure 6C), but had no effect on HK1 and HK2 (Figure 6, A–C). In control experiments, we found that the only other enzyme present in the cuvette (glucose-6-phosphate dehydrogenase) was not affected by either POA or BA (*P* > 0.6 and *P* > 0.4, respectively, as compared with control; *n* = 5). We also measured the activity of HK3, which has relatively low abundance in most tissues except myeloid cells, but we did not find any significant inhibition by either POA or BA as compared with control (*n* = 5). POAEE partially, but significantly, inhibited HK1 (*P* < 0.004, *n* = 3), but did not affect other HKs. Western blot (Figure 6D) showed that HK1, HK2, and HK4 were all present in mouse PACs. We conclude that pathological HK inhibition, particularly of HK1 by POA and HK4 by BA, plays a key role in the ATP depletion that is such an important feature of AP. In line with these data, a relatively high concentration of insulin (100 nM) stimulated HKs and alleviated asparaginase-, POA-, and BA-induced necrosis (Supplemental Figure 1D). An increased glucose concentration (30 mM) only stimulated glucokinase and, therefore, alleviated asparaginase-induced and POA-induced, but not BA-induced, necrosis (Supplemental Figure 1D).

### Galactose administration protects from alcohol-induced AP in vivo.

**Figure 7:**
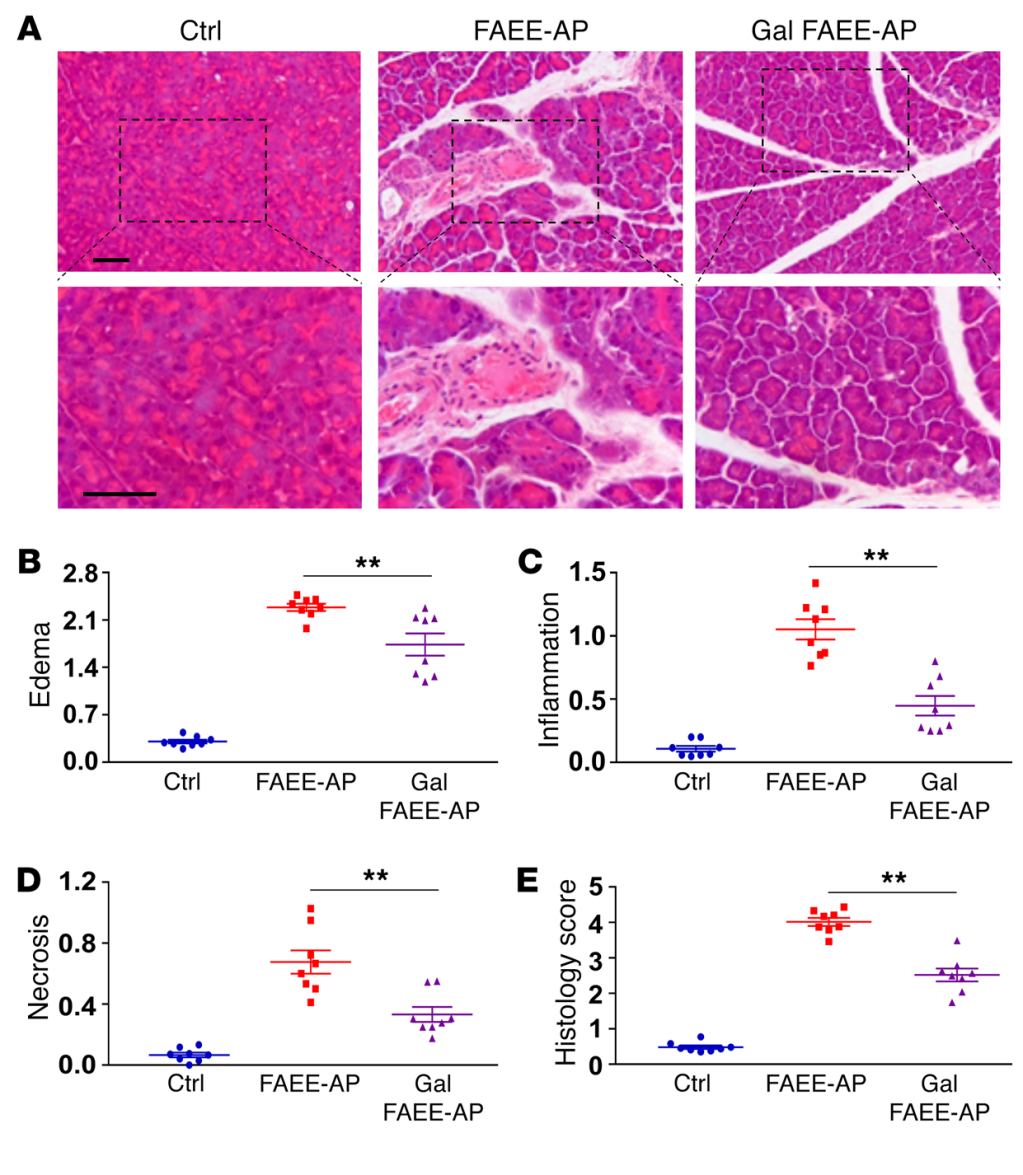
Galactose protects against alcohol-induced AP in vivo. (**A**) Galactose significantly improved the pathological scores in FAEE-AP. Representative H&E images of pancreas histology slides showing normal pancreatic histology (saline injection), and typical histopathology from FAEE-AP without or with galactose feeding (100 mM). Lower row of images shows zoomed parts of the images above. Scale bars: 50 μm. (**B**–**E**) Overall histopathological score (**E**) and components: edema (**B**), inflammation (**C**), and necrosis (**D**). All detrimental changes induced by POA and ethanol were significantly ameliorated by galactose (*P* < 0.007). Data are shown as mean ± SEM of 3 to 5 mice per group. **P < 0.01, 1-way ANOVA.

To determine whether our findings could lead to a rational treatment of AP, we focused our attention specifically on the possibility that galactose might be helpful, as this sugar has already been included as part of human trials for the treatment of glycogen storage disease type 1b (Fabry’s disease), nephrotic syndrome, and congenital disorders of glycosylation and has not been shown to have any negative effects (30–33). Galactose, an essential component of human breast milk (up to 70 mM during the first month; ref. 34), is quite stable in solution, relatively slowly metabolized compared with pyruvate, and has been administered both by i.p. injections and feeding (drink) protocols (35–37). We tested the protective effect of galactose in vivo in a realistic mouse model in which AP was induced by a mixture of POA and alcohol (FAEE-AP; ref. 38). As shown in Figure 7, A–E, galactose significantly improved the histology score (Figure 7E) and reduced the degrees of edema (Figure 7B), inflammation (Figure 7C), and necrosis (Figure 7D). Galactose also substantially reduced the alcohol-induced increase in amylase activity (Supplemental Figure 4A), IL-6, (Supplemental Figure 4B), and intracellular trypsin (Supplemental Figure 5, A–H). Control glucose feeding did not affect amylase activity (Supple mental Figure 4A), but was able to partially restore IL-6 levels. The weight loss typically seen in AP was partially prevented by galactose, but not glucose (Supplemental Figure 4, C and D). Overall, galactose had a remarkable protective effect against experimental alcohol-related AP.

### Galactose administration inhibits AAP in vivo.

The experiments shown in Figure 3, B and C, indicate that it might be possible to use galactose to boost energy production in vivo to counteract the toxic effects of asparaginase. We have therefore developed a mouse model of AAP using an approach similar to that developed for studying AP induced by alcohol metabolites, bile, and caerulein (38).

**Figure 8:**
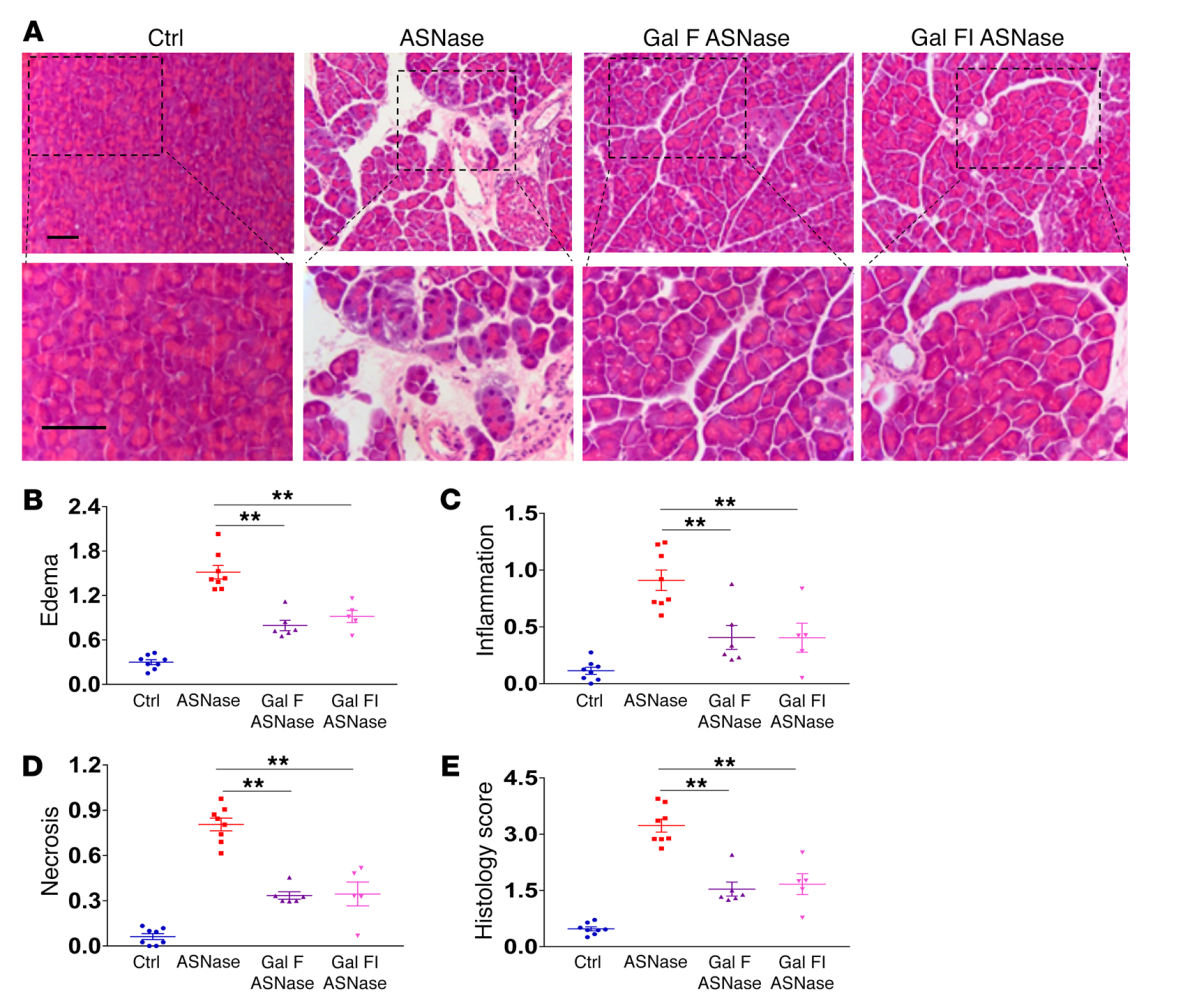
AAP is substantially reduced by galactose in vivo. (**A**) Representative H&E images of pancreas from slides showing normal pancreatic histology (saline), typical histopathology from AAP model (asparaginase 20 IU/g), and typical histopathology from treatment groups: galactose feeding (Gal F) and combination of galactose feeding and galactose injection (Gal FI). Lower row of images shows zoomed parts of the images above. Scale bars: 50 μm. (**B**–**E**) Edema (**B**), inflammation (**C**), necrosis (**D**), and overall histopathological score (**E**) in asparaginase-induced AP and the effects of the 2 different galactose treatment protocols. All detrimental changes induced by asparaginase were significantly ameliorated by galactose (*P* < 0.004; data are shown as mean ± SEM of 3–5 mice per group). **P < 0.01, 1-way ANOVA.

Asparaginase injections resulted in significantly increased histology scores and high degrees of edema, inflammation, and necrosis (Figure 8, A–E) that were similar to those reported for other AP models (38). As shown in Figure 8, A–E, galactose significantly reduced the histology score and the degrees of edema, inflammation, and necrosis toward much lower values in both protocols, feeding and a combination of injection and feeding, with similar efficacy. The weight loss typical for AP was also partially reduced (Supplemental Figure 4D). Therefore, we conclude that galactose could become an effective supplemental treatment for AAP.

## Discussion

It is well established that the initial stages of AP are characterized by intracellular Ca^2+^ overload, causing inadequate function of the mitochondria, leading to reduction of ATP production, premature intracellular activation of digestive enzymes, and cell death, mainly by necrosis (1, 2).

Our new data reveal that AP-inducing agents, such as alcohol and fatty acids, bile, and asparaginase, markedly reduce glucose metabolism in PACs, leading to reduced ATP synthesis and, therefore, substantial ATP loss. The combination of cytosolic Ca^2+^ overload and ATP depletion leads to profound cellular necrosis that could be avoided by ATP supplementation (22).

We have now shown that the addition of pyruvate or galactose substantially reduces cell injury induced by all the principal agents inducing AP. Removal of glucose from the medium does not significantly affect the ATP loss and necrosis induced by these agents, indicating that glucose metabolism is severely inhibited. Phloretin, the glucose transport inhibitor (39), also completely blocked the galactose rescue effect (Supplemental Figure 1C). Glucose and galactose are known to enter the cells by the same transporters (40), but galactose is converted to glucose-6-phosphate by several enzymes without involving HKs (41, 42). We therefore conclude that HK inhibition is likely to play an important role in the ATP depletion that is an important element in the development of AP.

Our in vitro experiments (Figure 6) suggest that both POA and BAs directly affect HK enzymes, HK1 and HK4, respectively, whereas the asparaginase effect is indirect (18). The direct inhibition of HKs reduces, but does not abolish, ATP production (Supplemental Figure 6, A–C), as there can still be some production by a number of metabolic pathways. However, cellular ATP is severely depleted, and at the same time, cells are overstimulated by pathological substances, making recovery virtually impossible. Galactose addition in vivo (as well as pyruvate in vitro) protects the cells from ATP depletion and hence necrosis.

A relatively high dose (100 nM) of insulin reduced all POA- (26), BA-, and asparaginase-induced necrosis (Supplemental Figure 1D), most likely by potentiating HKs (28, 29). An increased glucose concentration (30 mM) potentiated glucokinase, which has a low affinity for glucose (29), and also reduced both POA- and asparaginase-induced necrosis (Supplemental Figure 1D). However, such an increased glucose level failed to reduce BA-induced necrosis (Supplemental Figure 1D). This is in line with our data regarding the inhibition of glucokinase by BA (Figure 6C), whereas both POA and asparaginase have striking similarities in their pathological mechanisms, likely inhibiting HK1 (Figure 6A). Although both insulin and high glucose levels were effective in vitro, none of them could of course be employed in vivo. In contrast, galactose feeding, which appears to have no negative side effects, would be a potentially valuable therapy against AP.

Galactose could also be used preventively, which could be of particular importance in cases in which there is a significantly enhanced risk of AP (43), for example, when treating ALL with asparaginase. Our results indicate that galactose would be a valuable addition to the current asparaginase treatment protocol. Substitution of drinking water in mouse models with a 100 mM galactose solution markedly reduced all pathological scores in both asparaginase- and alcohol metabolite–induced AP. Since this approach has been successful in treating experimental AP induced by several different agents, i.e., asparaginase and POA, and relies on increasing intracellular ATP, preventing depletion of ATP, it might also become useful for treating other diseases with ATP loss and subsequent necrosis as well as counteracting similar side effects of other drugs.

With regard to the clinical treatment of patients with AP, there is currently a debate about high- versus low-energy administration in the early phase of AP (44). The protocol for a current multicenter, randomized, double-blind clinical trial only deals with the question of the potential merit of high-energy enteral tube feed versus zero-energy enteral tube feed (44). Our new results now suggest a need for clinical trials potentially using galactose instead of glucose in enteral tube feeds for patients in the early phase of AP.

## Methods

### Chemicals and reagents.

Fluorescent dyes Fluo-4-AM, MgGreen AM, and propidium iodide (PI) were purchased from Thermo Fisher Scientific. Collagenase was obtained from Worthington, asparaginase was purchased from Abcam, and POAEE was from Cayman Chemical. All other reagents were purchased from Sigma-Aldrich. C57BL/6J mice were obtained from The Jackson Laboratory.

### Antibodies.

Primary antibodies were as follows: anti-HK1 mouse monoclonal antibody (clone 7A7, catalog MA5-15675, 1/500; Thermo Fisher Scientific), anti-HK2 mouse monoclonal antibody (clone 1E8-H3-F11, catalog ab131196, 1/500; Abcam); anti-HK4 (GCK) rabbit polyclonal antibody (catalog PA5-15072, 1/500; Thermo Fisher Scientific); and anti–β-actin mouse polyclonal antibody (catalog sc-47778, 1/500; Santa Cruz Biotechnology Inc.). Secondary antibodies were as follows: Pierce goat anti-rabbit IgG, (H+L) peroxidase-conjugated antibody (catalog 31460 1/5,000; Thermo Fisher Scientific); and goat anti-mouse IgG-HRP (catalog sc-2005, 1/1000; Santa Cruz Biotechnology Inc.).

### Isolation of PACs.

Cells were isolated as previously described (18). After dissection, the pancreas was digested using collagenase-containing solution (200 IU/ml, Worthington) and incubated in a 37°C water bath for 14 to 15 minutes. The extracellular solution contained the following: 140 mM NaCl, 4.7 mM KCl, 10 mM HEPES, 1 mM MgCl_2_, 10 mM glucose, pH 7.3, and 1 mM CaCl_2_. Osmolarity was checked by Osmomat 030. All in vitro experiments were conducted using this solution unless otherwise stated.

### Fluorescence measurements.

For measurements of [Ca^2+^]_i_, isolated PACs were loaded with Fluo-4-AM (5 μM; excitation, 488 nm; emission, 510–560 nm) following the manufacturer’s instructions. Measurement of intracellular ATP was performed with MgGreen, which senses changes in [Mg^2+^]_i_ at concentrations around the resting [Mg^2+^]_i_ (18). PACs were incubated with 4 μM MgGreen AM for 30 minutes at room temperature (excitation, 488 nm; emission, 510–560 nm). ATP depletion mixture (4 μM CCCP, 10 μM oligomycin, and 2 mM iodoacetate) was applied for a final 10 minutes of each experiment to induce maximum ATP depletion (21). Asparaginase was used in a concentration of 200 IU/ml, 500 μM POAEE (from the stock solution in ethanol, Cayman Chemical), 50 μM POA (from 30 mM stock in ethanol), and 0.06% sodium choleate (BA) unless stated otherwise.

Necrotic cell death was assessed with PI uptake as previously described (excitation, 535 nm; emission, 617 nm) (4). The total number of cells showing PI uptake was counted in a series of 3 or more experiments for each treated group (>100 cells per each sample) to provide a percentage as the mean ± SEM.

All experiments were performed at room temperature using freshly isolated cells attached to coverslips of the perfusion chamber. Fluorescence was imaged over time using Leica SP5 2-photon, Leica TCS SPE, and Zeiss spin-disk confocal microscopes.

### In vivo models of asparaginase- and fatty acid ethyl ester–induced AP.

Before and throughout the experiment, unless otherwise noted, mice were maintained in plastic cages with corn cob bedding; tap water and commercial pelleted diet were freely provided. To establish AAP, C57BL6/J mice received 4 daily (24 hours apart) i.p. injections of asparaginase in PBS at 20 IU/g. Control mice received PBS-only i.p. injections. Treatment groups were defined as follows: (a) galactose-fed (100 mM in drinking water 24 hours before the first i.p. asparaginase and all the following days during injections) followed by asparaginase injection (20 IU/g) or (b) galactose-fed (100 mM galactose in drinking water) with i.p. galactose (180 mg/kg/d) and asparaginase (20 IU/g) (*n* = 5–8 mice/group). Mice were sacrificed 96 hours after first injection, and pancreas was extracted for histology or isolation of PACs. Blood was also collected for amylase and IL-6 measurements.

In the FAEE-induced AP (FAEE-AP) group, mice received 2 i.p. injections of ethanol (1.35 g/kg) and POA (150 mg/kg) at 1-hour intervals as previously described (38). The treatment group animals were fed with galactose (180 mg/kg/d) as described previously. Animals were sacrificed at 24 hours after the final injection.

### Histology.

Pancreatic tissue was fixed in 4% formaldehyde and embedded in paraffin. Histological assessment was performed after H&E staining of fixed pancreatic slices (4 μm thickness). Evaluation was performed on 10 or more random fields (magnification, ×200) by 2 blinded independent investigators grading (scale, 0–3) edema, inflammatory cell infiltration, and acinar necrosis as previously described (38), calculating the mean ± SEM (*n* = 3–5 mice/group).

### HK activity.

To assay inhibitory effects of POA and BA on the activity of HK1, HK2 (Novus Biological), and HK4 (Enzo Life Sciences), NADH generated by glucose-6-phosphate dehydrogenase was detected at 340 nm as described in the manufacturer’s protocols for the Hexokinase Assay Kit (MAK091, Sigma-Aldrich).

### Western blotting.

Equal amounts of proteins were resolved by sodium dodecyl sulfate–polyacrylamide gel electrophoresis (4%–12% SDS Bis Tris gels, Thermo Fisher Scientific) and blotted; membranes were probed with primary and then secondary antibodies.

### Measurements of mitochondrial membrane potential.

For measurements of mitochondrial membrane potential (Δψm) in PACs, we used the dequench mode, as previously described (25). Freshly isolated pancreatic cells were loaded with 20 μM tetramethylrhodamine methyl ester (TMRM) for 25 minutes at room temperature. Cells were then washed and resuspended in extracellular solution. Fluorescence was excited by a 535 nm argon laser line, and emission was collected above 560 nm. All experiments were conducted by using a Leica TCS SPE confocal microscope with a ×63 oil immersion objective. The region of interest for analyzing the change of Δψm was the whole cell.

### Measurements of mitochondrial Ca^2+^.

For mitochondrial calcium [Ca^2+^] measurements (45), freshly isolated PACs were loaded with 10 μM Rhod-2-AM for 48 minutes at 30°C. After incubation, the cells were centrifuged for 1 minute and resuspended in extracellular solution. The fluorescence of Rhod-2 was excited using a 535 nm laser line, and the emitted light was collected above 560 nm.

### Enzyme activity and IL-6 measurements.

Serum amylase was determined by spectrophotometer measurements at 405 nm (Jenway) using the Amylase Activity Assay Kit (MAK009, Sigma-Aldrich) according to the manufacturer’s instructions.

For visualization of trypsin activity, PACs were incubated in extracellular solution containing 10 μM rhodamine 110, bis-(CBZ-L-isoleucyl-l-prolyl-l-arginine amide) dihydrochloride (BZiPAR) (Molecular Probes, Thermo Fisher Scientific) (4), according to the manufacturer’s instructions. BZiPAR was excited with a 488 nm laser line; emission was collected at 508–530 nm.

IL-6 levels were determined by enzyme-linked immunosorbent assay (Abcam).

### ATP measurements.

Isolated PACs were incubated for 2 hours with either POA, BA, or asparaginase with appropriate controls. Cellular ATP was determined in a homogenized cell preparation using the ATP Assay Kit (Sigma-Aldrich) according to the manufacturer’s instructions.

### Statistics.

Data are presented as mean ± SEM. Statistical significance and *P* values were calculated using Student’s 2-tailed *t* test or ANOVA, with *P* < 0.05 and *P* < 0.01 considered statistically significant and *P* < 0.001 considered highly significant.

### Study approval.

All animal studies were ethically reviewed and conducted according to the United Kingdom Animal (Scientific Procedures) Act of 1986, approved by the United Kingdom Home Office. Animal procedures and experimental protocols were approved by the Animal Care and Ethics Committees at the Cardiff School of Biosciences.

## Author contributions

SP, JVG, TMT, OG, SS, OHP, and OVG designed the study. SP, JVG, TMT, OG, and OVG conducted and analyzed experiments. SP, JVG, OHP, and OVG wrote the manuscript. All authors read and approved the final draft of the manuscript.

## Supplementary Material

Supplemental data

## Version 1 06/12/2018

In-Press Preview

## Version 2 07/30/2018

Electronic publication

## Version 3 07/31/2018

Correction made to affiliations.

## Version 4 08/31/2018

Print issue publication
