# Supplementary material for: Galactose protects against cell damage in mouse models of acute pancreatitis
Source: J Clin Invest. 2018 Jul 30;128(9):3769–78. doi: 10.1172/JCI94714 (PMC6118583; doi:10.1172/JCI94714)
Supplement: Supplemental data [file jci-128-94714-s062.pdf]

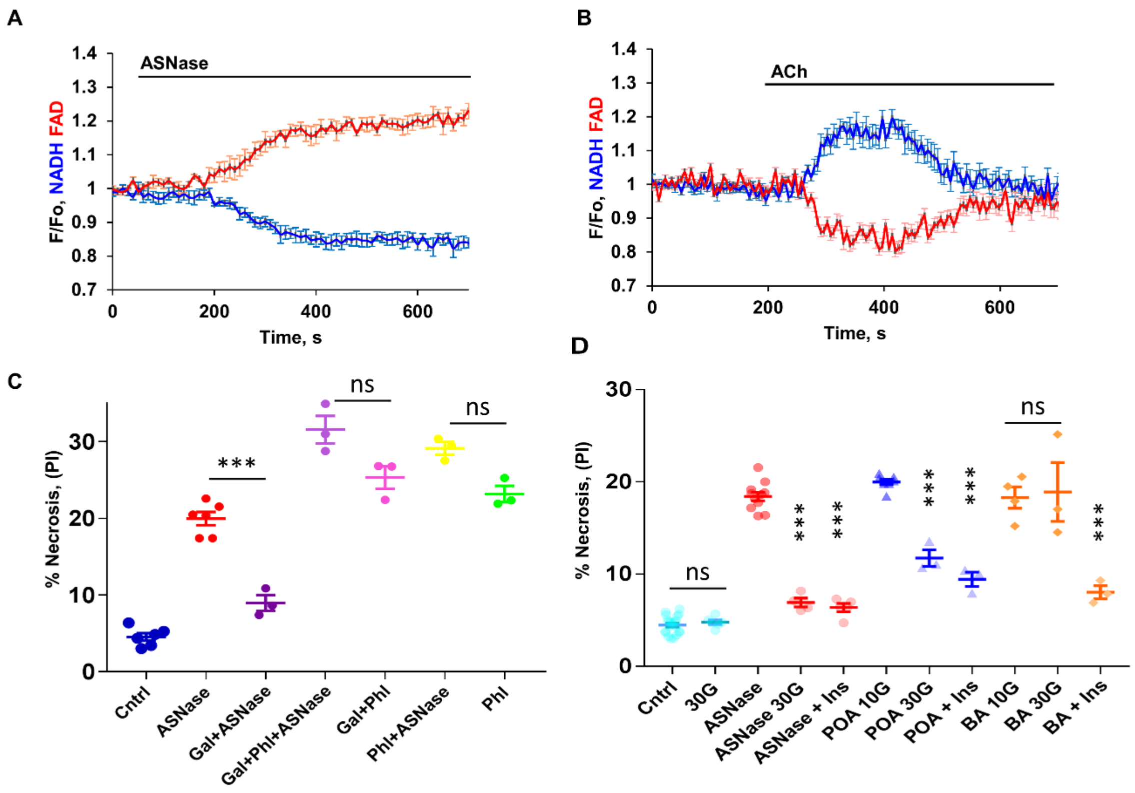


**Supplemental Figure 1. Asparaginase induced reduction in NADH and increase in FAD in PACs as opposed to ACh. Phloretin inhibits both glucose and galactose transport. 30 mM glucose rescues only Asparaginase- and POA-induced necrosis, whereas 100 nM insulin rescued the necrosis induced by all three agents, Asparaginase, POA and BA.**

A. Average traces represent simultaneous measurements of cytosolic NADH and FAD in PACs during application of 200 IU/ml ASNase. Bars present mean±SEM (n=8).

B. Average traces represent simultaneous measurements of cytosolic NADH and FAD in PACs during application of 100 nM ACh. Bars present mean±SEM (n=8).

C. Comparison of necrotic cell death levels induced by 2 hours incubation of PACs with 200 IU/ml ASNase in the absence or presence of 1 mM Galactose or 1 mM phloretin (inhibitor of active glucose transport into cells) (PI stained cells, p = 0.36, three series of experiments with n>100 cells in each sample).

D. Comparison of ASNase- POA- and BA-induced necrosis in different conditions: increased glucose (30mM) and in the presence of insulin (100nM). Increased glucose (30 mM) on its own did not change the control level of necrosis (p>0.4), but reduced significantly the level of necrosis induced by ASNase and POA (p<0.0001). In contrast, 30 mM glucose did not change the level of necrosis induced by BA (p>0.8). 100nM of insulin reduced significantly the level of necrosis induced by ASNase (p<0.0001), POA-(p<0.0001) and BA (p<0.001) (PI stained cells, dots represent series of experiments with n>100 cells in each sample).

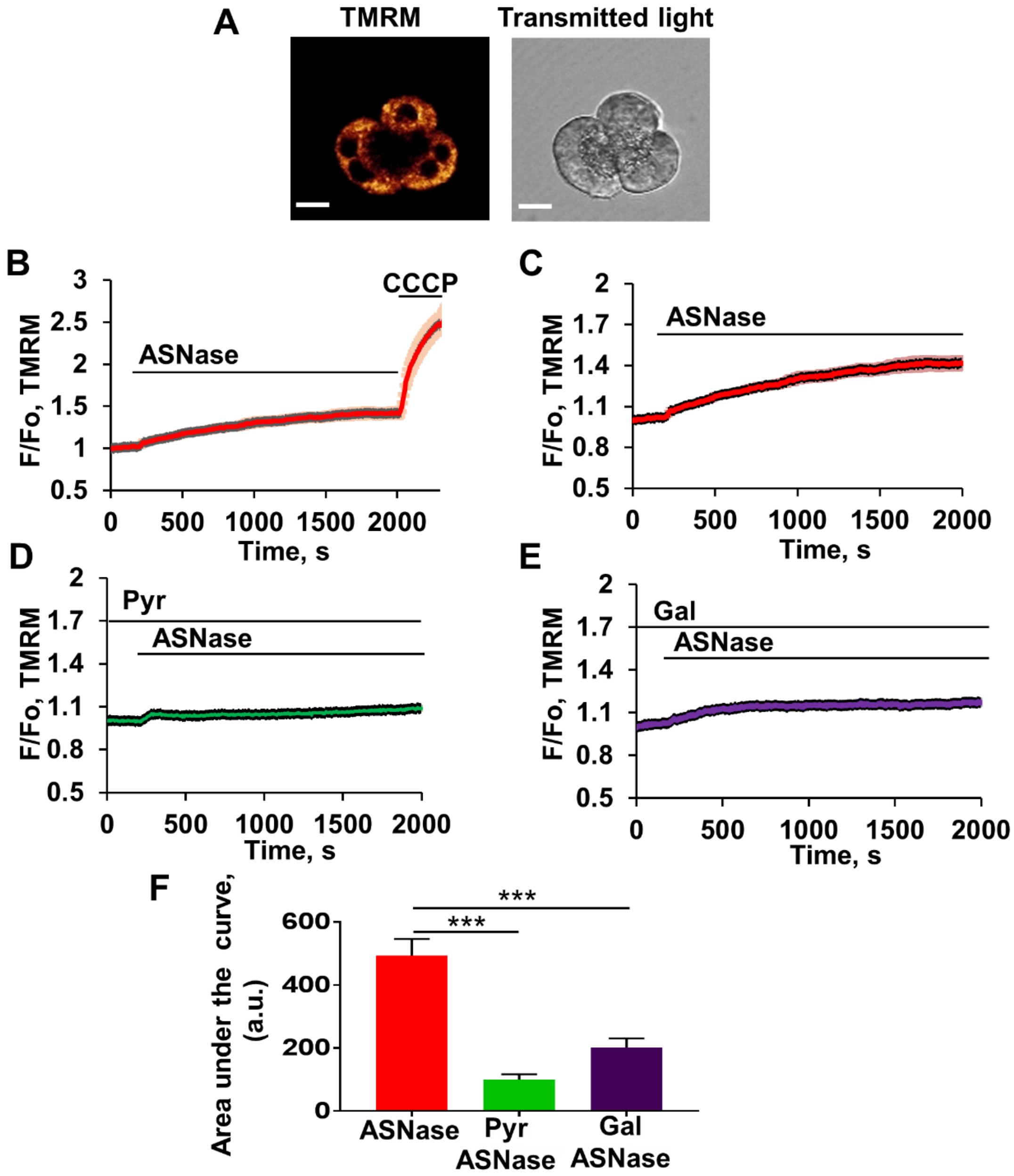


**Supplemental Figure 2. Asparaginase-induced mitochondrial depolarisation is rescued by pyruvate or galactose.**

A. TMRM fluorescence and transmitted light image of the cell cluster. (Scale bar: 10 μm)
B. Measurements of the mitochondrial membrane potential were performed using the fluorescence probes TMRM. The effect of ASNase (200 IU/ml)-induced loss of ΔψM followed by 5 nM CCCP on mitochondrial membrane potential in pancreatic acinar cells loaded with 10 μM TMRM (dequench mode, n = 33).
C. The trace shows normalized fluorescence changes induced by ASNase (200 IU/ml) in pancreatic acinar cells within 30 min (n = 33).
D. The ASNase effect was markedly reduced by 5 min incubation of 1mM pyruvate in pancreatic acinar cells (n = 12).
E. The ASNase effect was also markedly reduced by 15 min incubation 1mM galactose in pancreatic acinar cells (n = 12).
F. Comparisons of the integrated responses show that pyruvate and galactose significantly reduced the ASNase-induced mitochondrial depolarization ($p < 0.001$ for both treatments).

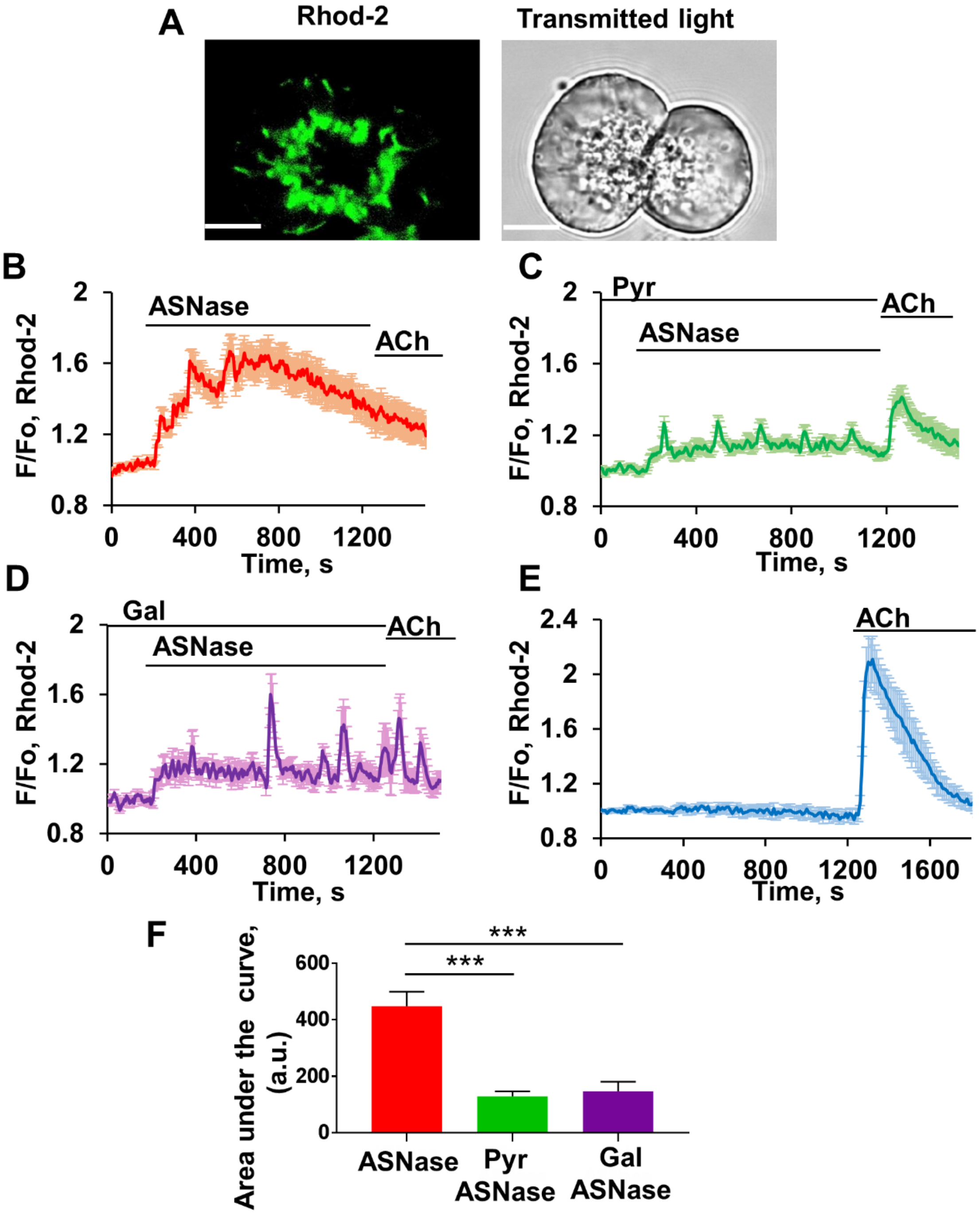


**Supplemental Figure 3. Asparaginase-induced mitochondrial calcium overload is rescued by pyruvate or galactose.**

A. Rhod-2 fluorescence and transmitted light image of doublet of pancreatic acinar cells. (Scale bar: 10 µm)

B. Measurements of the mitochondrial calcium were performed using the fluorescence probes Rhod-2-AM. The trace shows effects of ASNase (200 IU/ml) followed by 1 µM ACh on mitochondrial calcium (n = 14).

C. The trace shows effect of ASNase (200 IU/ml) on mitochondrial calcium response significant is decreased by 5 min preincubation of 1 mM pyruvate in pancreatic acinar cells (n = 17).

D. The trace shows effect of ASNase (200 IU/ml) on mitochondrial calcium response significant is decreased by 15 min preincubation of 1 mM galactose in pancreatic acinar cells (n = 6).

E. The trace shows mitochondrial calcium response by 1 µM ACh (n = 7).

F. Quantitative analysis of experiments of the type shown in B-D by comparing the integrated mitochondrial calcium change above the baseline (area under the curve) recorded during the first 1000 sec of ASNase application (p < 0.001 for both treatments).

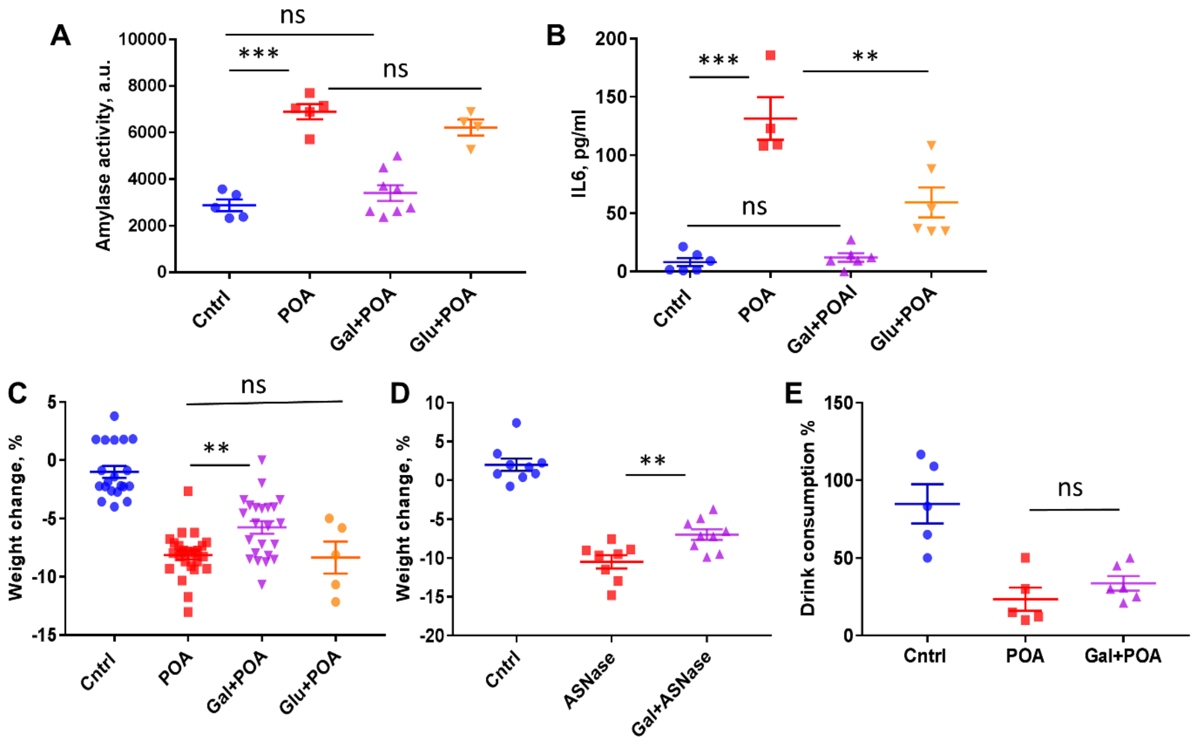


**Supplemental Figure 4. Galactose but not glucose feeding has rescued blood amylase levels and IL6 in the alcohol-induced AP model.**

**A.** Plasma amylase levels have been substantially elevated in alcohol-induced AP model ($p<0.0001$, n=5). Galactose feeding has reduced amylase levels to nearly control levels ($p>0.5$, n=8). Glucose feeding (180mg/kg/day) did not significantly change amylase levels ($p>0.5$, n=4).

**B.** Plasma IL6 levels have been substantially increased in alcohol-induced AP model ($p<0.0001$, n=4). Galactose feeding has reduced IL6 levels to nearly control levels ($p>0.9$, n=6). Glucose feeding has partially reduced elevated IL6 levels ($p<0.006$, n=6).

**C.** Weight loss typical for the AP model ($p<0.0001$, n=26) has been partially reduced by galactose feeding ($p<0.006$, n=23). Glucose feeding did not significantly change weight loss ($p>0.9$, n=5).

**D.** Weight loss induced by Asparaginase model of AP ($p<0.0001$, n=8) has been partially reduced by galactose ($p<0.01$, n=9).

**E.** Drink consumption has been reduced in alcohol-induced AP model ($p<0.0007$, n=5). Galactose feeding has not significantly change reduced drink consumption ($p>0.6$, n=6).

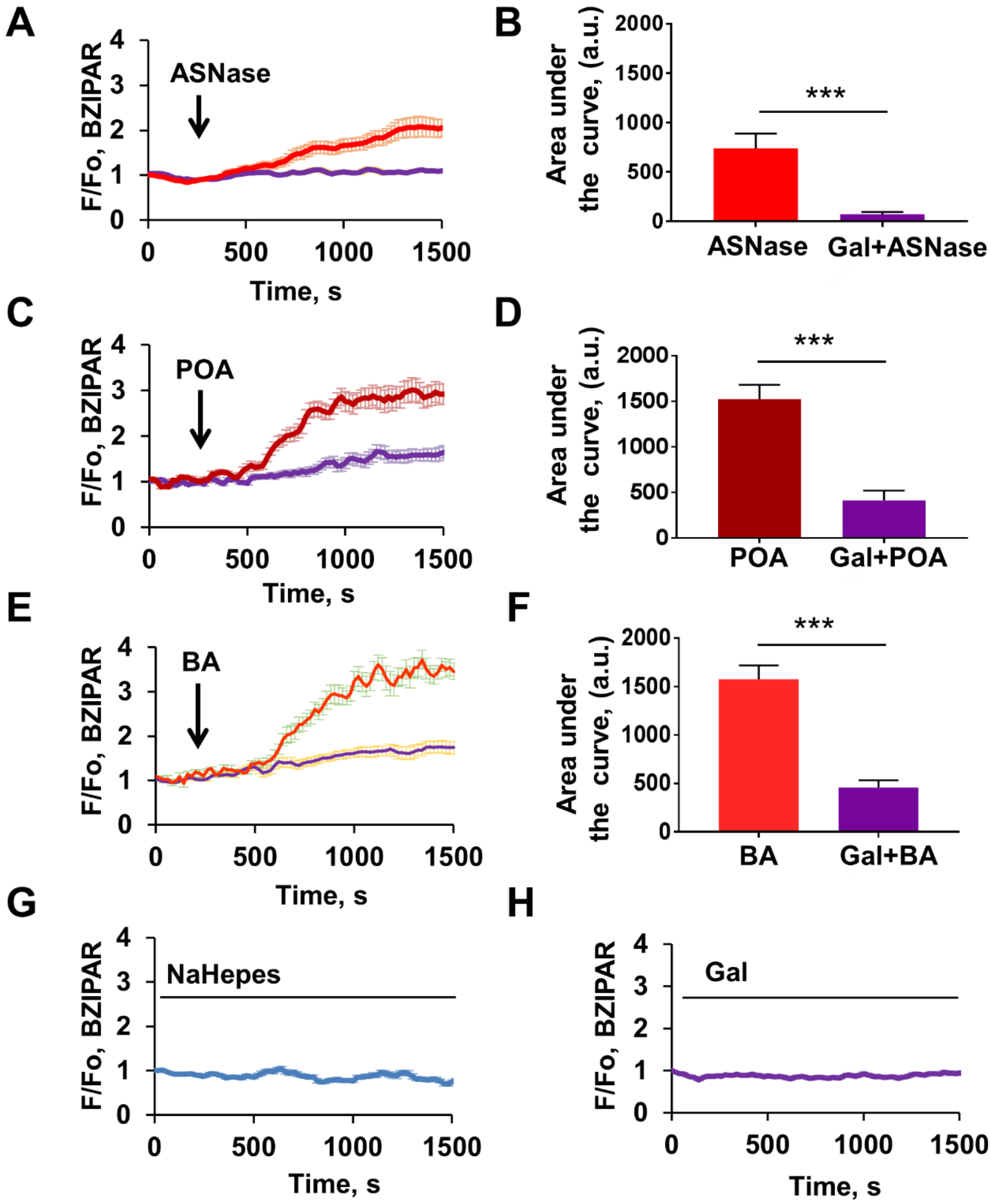


**Supplemental Figure 5. Intracellular trypsin activation induced by Asparaginase, POA or BA has been substantially reduced by galactose.**

**A.** ASNase (200 IU/ml) – evoked trypsin activation was reduced by 10 mM galactose. Averaged traces with error bars (ASNase, n=12; ASNase + Galactose, n=15).

**B.** 'Area under the curve' comparison of traces shown in A. Galactose significantly reduced ASNase- evoked trypsin activation (p<0.0001).

**C.** POA (50µM) –evoked trypsin activation was substantially reduced by 10 mM galactose. Averaged traces with error bars (POA, n=16; POA + Galactose, n=25).

**D.** 'Area under the curve' comparison of traces shown in C. Galactose markedly reduced POA-induced ATP depletion (p<0.0001).

**E.** BA (0.06%) –evoked trypsin activation is reduced by 10 mM galactose. Averaged traces with error bars (BA, n=12; BA + Galactose, n=22).

**F.** 'Area under the curve' comparison of traces shown in E. Galactose markedly reduced POA-induced ATP depletion (p<0.0001).

**G.** Averaged trace shows BZiPAR fluorescence of non-stimulated control in pancreatic

acinar cells (n=17).

**H.** Averaged trace shows BZiPAR fluorescence of 10 mM galactose treatment in pancreatic acinar cells (n=18).

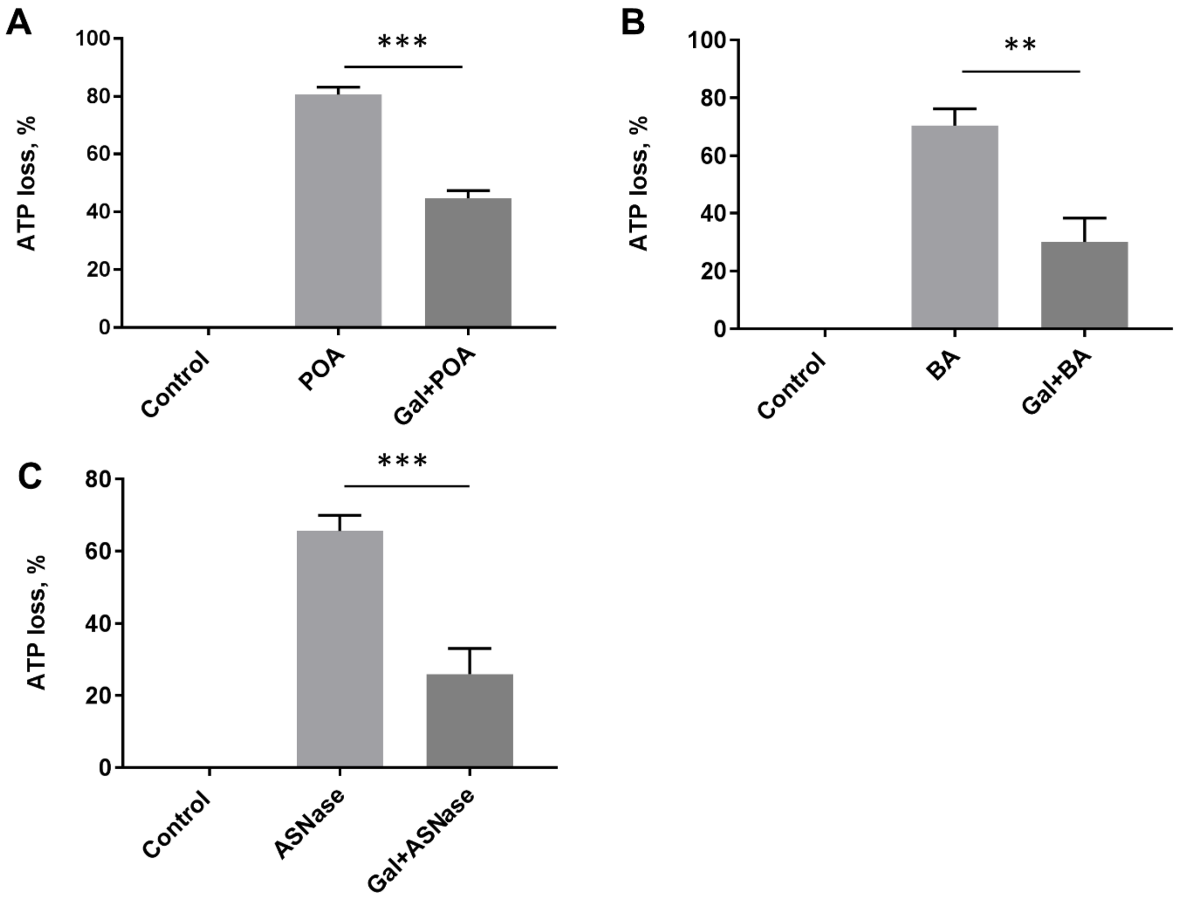


**Supplemental Figure 6.**

**Galactose partially rescues ATP depletion in cells treated with either POA or BA or Asparaginase.**

- **A.** POA treatment (2 hours) induced a substantial ATP loss in PACs ($p<0.0001$, n=4). Galactose substantially reduced ATP loss ($p<0.0001$, n=4).
- **B.** BA treatment of PACs induced a substantial ATP loss ($p<0.0001$, n=4). Galactose substantially reduced ATP loss ($p<0.0022$, n=4).
- **C.** Asparaginase treatment of PACs induced a substantial ATP loss ($p<0.0001$, n=4). Galactose substantially reduced ATP loss ($p<0.0006$, n=4).
